# Chronic Overlapping Pain Conditions in people with Myalgic Encephalomyelitis/Chronic Fatigue Syndrome (ME/CFS): a sample from the Multi-site Clinical Assessment of ME/CFS (MCAM) study

**DOI:** 10.1186/s12883-024-03872-0

**Published:** 2024-10-18

**Authors:** Elizabeth A. Fall, Yang Chen, Jin-Mann S. Lin, Anindita Issa, Dana J. Brimmer, Lucinda Bateman, Charles W. Lapp, Richard N. Podell, Benjamin H. Natelson, Andreas M. Kogelnik, Nancy G. Klimas, Daniel L. Peterson, Elizabeth R. Unger, for the MCAM Study Group, Elizabeth A. Fall, Yang Chen, Jin-Mann S. Lin, Lucinda Bateman, Charles W. Lapp, Richard N. Podell, Benjamin H. Natelson, Andreas M. Kogelnik, Nancy G. Klimas, Daniel L. Peterson, Elizabeth R. Unger, Hao Tian, Kathleen Bonner, Monica Cornelius, Irina Dimulescu, Britany Helton, Maung Khin, Mangalathu Rajeevan, Jennifer Bland, Patricia Jeys, Veronica Parkinson, Wendy Springs, Elizabeth Balbin, Jeffry Cournoyer, Melissa Fernandez, Shuntae Parnell, Precious Leaks-Gutierrez, Michelle Blate, Gudrun Lange, Sarah Khan, Diana Vu, Joan Danver, David Kaufman, Macy Pa, Catt Phan, Sophia Taleghani, Trisha Fitzpatrick, Beverly Licata, Elena Lascu, Gunnar Gottschalk, Marco Maynard

**Affiliations:** 1 Division of High-Consequence Pathogens and Pathology, National Center for Emerging and Zoonotic Infectious Diseases, Centers for Disease Control and Prevention, Atlanta, GA USA; 2 Bateman Horne Center, Salt Lake City, UT USA; 3 Hunter-Hopkins Center, Charlotte, NC USA; 4 Richard N. Podell Medical, Summit, NJ USA; 5 Department of Neurology, Icahn School of Medicine at Mount Sinai, New York, NY USA; 6 Basis Diagnostics, Redwood City, CA USA; 7 Institute for Neuro Immune Medicine, Nova Southeastern University, Fort Lauderdale, FL USA; 8 Geriatric Research and Education Clinical Center, VA Medical Center, Miami, FL USA; 9 Sierra Internal Medicine, Incline Village, NV USA

**Keywords:** Myalgic encephalomyelitis/Chronic fatigue syndrome (ME/CFS), Multimorbidity, Chronic low back pain (cLBP), Chronic migraine/Headache (cMHA), Fibromyalgia (FM), Interstitial cystitis/Irritable bladder (IC/IB), Irritable bowel syndrome (IBS), Temporomandibular disorder (TMD), Endometriosis, Vulvodynia

## Abstract

**Background:**

Chronic overlapping pain conditions (COPCs), pain-related conditions that frequently occur together, may occur in patients with myalgic encephalomyelitis/chronic fatigue syndrome (ME/CFS) and could impact illness severity. This study aimed to identify comorbid COPCs in patients with ME/CFS and evaluate their impact on illness severity.

**Methods:**

We used data from 923 participants in the Multi-Site Clinical Assessment of ME/CFS study, conducted in seven U.S. specialty clinics between 2012 and 2020, who completed the baseline assessment (595 ME/CFS and 328 healthy controls (HC)). COPCs included chronic low back pain (cLBP), chronic migraine/headache (cMHA), fibromyalgia (FM), interstitial cystitis/irritable bladder (IC/IB), irritable bowel syndrome (IBS), temporomandibular disorder (TMD). Illness severity was assessed through questionnaires measuring symptoms and functioning. Multivariate analysis of variance and analysis of covariance models were used for analyses. Log-binomial regression analyses were used to compute prevalence of COPCs and prevalence ratios (PR) between groups with 95% confidence intervals. Both unadjusted and adjusted results with age and sex are presented.

**Results:**

76% of participants with ME/CFS had at least one COPCs compared to 17.4% of HC. Among ME/CFS participants, cMHA was most prevalent (48.1%), followed by FM (45.0%), cLBP (33.1%), and IBS (31.6%). All individual COPCs, except TMD, were significantly more frequent in females than males. The unadjusted PR (ME/CFS compared to HC) was highest for FM [147.74 (95% confidence interval (CI) = 20.83-1047.75], followed by cLBP [39.45 (12.73-122.27)], and IC/IB [13.78 (1.88-101.24)]. The significance and order did not change after age and sex adjustment. The COPC comorbidities of cLBP and FM each had a significant impact on most health measures, particularly in pain attributes (Cohen’s d effect size 0.8 or larger). While the impact of COPC comorbidities on non-pain attributes and quality of life measures was less pronounced than that on pain, statistically significant differences between ME/CFS participants with and without COPCs were still evident.

**Conclusions:**

More than 75% of ME/CFS participants had one or more COPCs. Multiple COPCs further exacerbated illness severity, especially among females with ME/CFS. Assessment and management of COPCs may help improve the health and quality of life for patients with ME/CFS.

**Supplementary Information:**

The online version contains supplementary material available at 10.1186/s12883-024-03872-0.

## Background

 Myalgic encephalomyelitis/chronic fatigue syndrome (ME/CFS) is a debilitating, long-term illness that affects multiple body systems. ME/CFS is defined by reduced ability to perform pre-illness activities, lasting 6 months or longer, that is accompanied by profound fatigue, which is not improved by rest and not a result of ongoing exhaustion [1]. A hallmark of ME/CFS is that symptoms can worsen after physical, mental, or emotional effort, a manifestation known as post-exertional malaise (PEM). Patients with ME/CFS also have unrefreshing sleep and either orthostatic intolerance, cognitive impairment, or both [1]. The frequency and severity of these symptoms vary by individual and potentially change over time. In addition, people with ME/CFS may experience a wide range of additional symptoms not used in the case definition because of their variability. Of these additional symptoms, those relating to pain are most common. Comorbid pain conditions can worsen symptom severity and overall health status, making this illness challenging to clinically manage [2–4].

Chronic overlapping pain conditions (COPCs) are a group of chronic pain conditions that often occur together and are thought to share similar disease mechanisms. People with one COPC have an increased likelihood of having another COPC [5]. Among people with more than one COPC, perception of pain is likely to be further amplified. A study of 1,413 migraineurs found that symptoms and severity of cutaneous allodynia were associated with pain comorbid conditions [6]. These pain disorders have been reported as comorbidities in people with ME/CFS [3]. Multiple studies have reported COPCs in people with ME/CFS including fibromyalgia (21–61% [2, 7–9]), irritable bowel syndrome (67% [10]), temporomandibular disorder (21–32% [11]), migraines/headaches (63-84% [12, 13]), and endometriosis (20.1–36% [2, 14, 15]). However, research is limited on the co-occurrence and functional impact of certain COPCs, such as interstitial cystitis/irritable bladder, chronic low back pain, and vulvodynia in people with ME/CFS. Furthermore, most of these studies have relatively small sample sizes, evaluated one or two COPCs, and used a past history of COPCs.

Studies have reported that ME/CFS with co-occurring fibromyalgia is associated with greater disease impact [16], more bodily pain [17], and worse physical functioning [18, 19] than ME/CFS alone. These findings suggest that severity of ME/CFS may be heightened in people with co-occurring COPCs. Multimorbidity further increases healthcare costs and the complexity of clinical management [20–22]. The higher level of pain and functional impairment associated with multiple COPCs necessitates a more comprehensive and tailored approach to treatment with a multidisciplinary team of healthcare providers.

This study aims to address the prevalence of COPCs in those with ME/CFS and their impact on symptom severity, functioning levels, and quality of life. While previous studies have examined one or a subset of COPCs in people with ME/CFS, there is still a need to explore how these conditions contribute to symptom burden, both individually and cumulatively. By addressing these gaps in our understanding, this study seeks to help inform healthcare professionals about the need for an individualized approach to clinical management of symptoms in ME/CFS, with the goal of reducing the effect of COPCs on illness severity in ME/CFS.

## Methods

### Data source and study sample

The primary data collection was from the Multi-Site Clinical Assessment of Myalgic Encephalomyelitis/ Chronic Fatigue Syndrome (MCAM) study [23, 24]. The study was reviewed and approved by the Institutional Review Boards of the Centers for Disease Control and Prevention (CDC), Open Medicine Institute Consortium, Mount Sinai Beth Israel, and Nova Southeastern University. One of the objectives for the source study, MCAM, was to improve how ME/CFS illness domains could be measured and detected in people with ME/CFS. In brief, MCAM was conducted between 2012 and 2020 in multiple stages with a rolling cohort design. Not all participants were enrolled in the same stage; therefore, baseline data could come from any stage. Participants were recruited from seven ME/CFS specialty clinics across seven states. The MCAM study relied on ME/CFS expert clinicians to determine patient eligibility for the ME/CFS group using their clinical expertise with the illness. Inclusion criteria were people 18–70 years of age who had been diagnosed with CFS, ME, or post-infectious fatigue or who were managed as other ME/CFS patients in the clinical practice. Healthy controls (HC) were recruited from the neighborhood of clinics through health screening events or flyers.

We used cross-sectional medical history data abstraction from 923 participants who completed the baseline assessment to investigate the burden of COPCs. This study sample consisted of 595 ME/CFS and 328 HC. We hypothesized that people with ME/CFS would have a higher prevalence of COPCs than healthy controls and that COPC comorbidities would increase the symptom burden and worsen functioning among those with ME/CFS, particularly in pain-related health measures.

### Measures

#### Chronic Overlapping Pain Conditions (COPCs)

The eight COPCs of interest in this paper include: chronic low back pain (cLBP), chronic migraine/headache (cMHA), fibromyalgia (FM), interstitial cystitis/irritable bladder (IC/IB), irritable bowel syndrome (IBS), temporomandibular disorder (TMD), and two female-only conditions: endometriosis and vulvodynia. The presence of each of the eight COPCs was determined from the Medical History form based on records of ongoing (current) COPCs with chronicity of 3 months or longer. The Medical History form abstracted information on review of organ systems, major illnesses, and age onset of these health problems [23]. To further ascertain cLBP, we utilized information from the Brief Pain Inventory (BPI) [25] full body map to identify participants who indicated the lower back as the site of their pain. A total number of COPCs for each participant was generated as an index. To make the index comparable for males and females, the two female-only COPCs were not included in the index calculation, resulting in an index range from 0 (none present) to 6 (all present).

#### Assessment of Health Measures

Health measures of study participants included three assessment tools: (1) ME/CFS-related symptoms: 19 ME/CFS-related symptom scores (CDC-SI) [26]; (2) other symptom-oriented domain measures: *Fatigue* assessed by Multidimensional Fatigue Inventory (MFI-20) [27, 28] and Patient-Reported Outcomes Measurement Information System (PROMIS) Fatigue Short Form [29, 30]; *Pain* assessed by Brief Pain Inventory [25] [BPI: Severity of Pain (SOP) and Interference of Pain (IOP)], PROMIS Pain Behavior Short Form [31, 32], and PROMIS Pain Interference Short Form [32]; and *Sleep* assessed by PROMIS Sleep Disturbance Short Form and PROMIS Sleep-Related Impairment [32, 34]; (3) function-oriented measures: Well-being and Functioning assessed by Short Form Health Survey (SF-36v2) [35]; and the CDC Health-Related Quality of Life (HRQoL-14) for physically and mentally unhealthy days [36].

The PROMIS and SF-36 instruments generate T-scores, in which a score of 50 represents the mean score of a reference population (usually the U.S. general population), with a Standard Deviation (SD) of 10. The T-score metrics have been established and validated for health measures. In general, higher scores indicate more of the concept being measured (e.g., greater fatigue for PROMIS Fatigue, higher functioning for SF-36 Physical Functioning).

Additionally, we included demographic information in the analysis including age, sex, race/ethnicity, marital status, employment, health insurance, education level, number of office visits over the past year, body mass index (BMI), illness duration, and onset status.

### Statistical Analysis

Descriptive statistics and outcome measures were shown as means and ± standard deviations (SD) for continuous variables and frequency counts and percentages for categorical variables. Comparisons between groups were evaluated using χ^2^ tests or Fisher’s exact tests when appropriate for categorical variables. The mean number of COPC categories along with p-values for differences between ME/CFS and HC groups, was determined using analysis of variance and analysis of covariance models. Log-binomial regression analyses were used to compute estimated prevalence of COPCs within each group and prevalence ratios (PR) between groups and their 95% confidence intervals (CI). Both unadjusted and adjusted results with age and sex covariates are presented. All analyses were conducted using SAS software, version 9.4 [37] and the level of significance was set at *p* < 0.05. For ease of visualization, we used the R ggplot2 package [38] to generate heatmap graphs for unadjusted mean differences in each health measure score between ME/CFS participants with and without COPCs. An UpSet plot was created using the UpSetR package [39] in R to show the combinations of COPCs experienced by ME/CFS participants.

## Results

Sample characteristics of the 923 participants by study group (ME/CFS vs. HC) are shown in Table 1. Participants with ME/CFS were older (47.6 vs. 42.8 years, *p* < 0.0001), more likely to be female (72.8% vs. 65.9%, *p* = 0.0276), White (88.2% vs. 57.0%, *p* < 0.0001), non-Hispanic (79.3% vs. 57.0%, *p* < 0.0001), and to be not currently working (69.7% vs. 25.3%, *p* < 0.0001), had health insurance (92.3% vs. 81.1%, *p* < 0.0001), had more office visits over the past year (4.7 vs. 0.6 visits, *p* < 0.0001), and a higher COPCs index score (1.8 vs. 0.2, *p* < 0.0001). No statistical difference was found in marital status, educational attainment, and obesity. Participants with ME/CFS had a mean illness duration of 13.6 years and the majority reported a sudden onset of illness (59.2%).

**Table 1:** Sample characteristics by Study Group (*n* = 923)

	ME/CFS (*n* = 595)	HC (*n* = 328)	*p*-value
Age (yrs), mean (SD)	47.6 (12.8)	42.8 (14.7)	< 0.0001
Female, n (%)	433 (72.8)	216 (65.9)	0.0276
Race^a^, n (%)			< 0.0001
White	525 (88.2)	187 (57.0)	
Black/African American	12 (2.0)	22 (6.7)	
All others	28 (4.7)	85 (25.9)	
Ethnicity^a^, n (%)			< 0.0001
Hispanic	39 (6.6)	94 (28.7)	
Non-Hispanic	472 (79.3)	187 (57.0)	
Marital Status^a^, n (%)			0.9047
Married/committed	308 (51.8)	164 (50.0)	
Previously married	103 (17.3)	56 (17.1)	
Never married	171 (28.7)	99 (30.2)	
Employment^a^, n (%)			< 0.0001
Full-time	89 (15.0)	177 (54.0)	
Part-time	65 (10.9)	58 (17.7)	
Not working	415 (69.7)	83 (25.3)	
Had insurance, n (%)	549 (92.3)	266 (81.1)	< 0.0001
Education^a^, n (%)			0.1299
Less than high school	4 (0.7)	5 (1.5)	
High school graduate	131 (22.0)	94 (28.7)	
College graduate	234 (39.3)	119 (36.3)	
Post college	208 (35.0)	102 (31.1)	
Number of office visit, mean (SD)	4.7 (12.86)	0.6 (1.24)	< 0.0001
BMI, mean (SD)	26.6 (6.12)	26.1 (5.48)	0.2602
Obesity, n (%)	111 (18.7)	55 (16.8)	0.2226
Illness Duration (yrs), mean (SD)	13.6 (10.25)		
Illness Onset^a^, n (%)			
Gradual	205 (34.5)		
Sudden	352 (59.2)		
COPC Index Score (0–6), mean (SD)	1.8 (1.45)	0.2 (0.43)	< 0.0001
Number of COPCs, n (%)			< 0.0001
0	142 (23.9)	271 (82.6)	
1	151 (25.4)	52 (15.9)	
2	123 (20.7)	5 (1.5)	
3 or more	179 (30.0)	0 (0)	

SD = Standard deviation; BMI = Body mass index; COPCs = Chronic overlapping pain conditions

^a^The frequency and percentage of missing values are not shown; therefore the total percentage for the indicated categories are not summed up to 100%

### Prevalence and Prevalence ratios of COPCs Comorbidities

Prevalence and prevalence ratios (PR) of individual COPCs (excluding female-only COPCs) are presented in Table 2. Over three-quarters (76.1%) of participants with ME/CFS had at least one of six COPCs while only 17.4% HC participants had one (15.9%) or two (1.5%) COPCs. Prevalence of all individual COPCs was significantly higher in ME/CFS than HC. Among ME/CFS participants, cMHA was the most prevalent COPC (48.1%), followed by FM (45.0%), cLBP (33.1%), and IBS (31.6%). Prevalence of all individual COPCs, except TMD, was significantly higher in females than males. Those with FM or/and cLBP were notably older than those without (Table S1). Female-only COPCs were less common in both ME/CFS and HC female participants compared to other COPCs (endometriosis: ME/CFS: 5.3%, HC: 2.3%, *p* = 0.0766; vulvodynia: ME/CFS: 0.7%, HC: 0.5%, *p* = 1.0000). The unadjusted PR (ME/CFS compared to HC) was highest for FM [147.74 (95% confidence interval (CI) = 20.83-1047.75], followed by cLBP [39.45 (12.73-122.27)], and IC/IB [13.78 (1.88-101.24)]. After adjustment for age and sex, the PRs remained significantly different with the same order.

**Table 2:** Prevalence and Prevalence Ratios for COPCs between ME/CFS (*n* = 595) and HC (*n* = 328)

			Unadjusted		Age Adjusted		Age and Sex Adjusted	
		%	PR	95% CI	PR	95% CI	PR	95% CI
cMHA								
ME/CFS	286	48.1	^d^4.15	3.04–5.66	^d^4.28	3.14–5.84	^d^4.19	3.07–5.72
HC	38	11.6	1.00	Referent	1.00	Referent	1.00	Referent
FM								
ME/CFS	268	45.0	^d^147.74	20.83-1047.75	^d^138.12	19.47-979.75	^d^133.40	18.81-946.07
HC	1	0.3	1.00	Referent	1.00	Referent	1.00	Referent
cLBP								
ME/CFS	197	33.1	^d^39.45	12.73-122.27	^d^37.15	11.98-115.21	^d^36.23	11.69-112.33
HC	3	0.9	1.00	Referent	1.00	Referent	1.00	Referent
IBS								
ME/CFS	188	31.6	^d^6.91	4.16–11.49	^d^6.84	4.10-11.39	^d^6.76	4.06–11.25
HC	15	4.6	1.00	Referent	1.00	Referent	1.00	Referent
TMD								
ME/CFS	82	13.8	^d^11.30	4.18–30.55	^d^10.98	4.05–29.76	^d^10.72	3.95–29.07
HC	4	1.2	1.00	Referent	1.00	Referent	1.00	Referent
IC/IB								
ME/CFS	25	4.2	^b^13.78	1.88-101.24	^a^12.11	1.64–89.14	^a^11.16	1.51–82.18
HC	1	0.3	1.00	Referent	1.00	Referent	1.00	Referent
Any COPCs								
ME/CFS	453	76.1	^d^4.58	3.60–5.82	^d^4.48	3.53–5.70	^d^4.38	3.45–5.57
HC	57	17.4	1.00	Referent	1.00	Referent	1.00	Referent

*PR* = prevalence ratio, *95% CI* = 95% confidence interval

*cMHA* = chronic migraine/headache, *FM* = fibromyalgia, *cLBP* = chronic low back pain, *IBS* = irritable bowel syndrome, *TMD* = temporomandibular disorder, *IC/IB* = interstitial cystitis/irritable bladder, *COPCs* = chronic overlapping pain conditions

^a^*p* < 0.05

^b^*p* < 0.01

^c^*p* < 0.001

^d^*p* < 0.0001

**Fig. 1:**
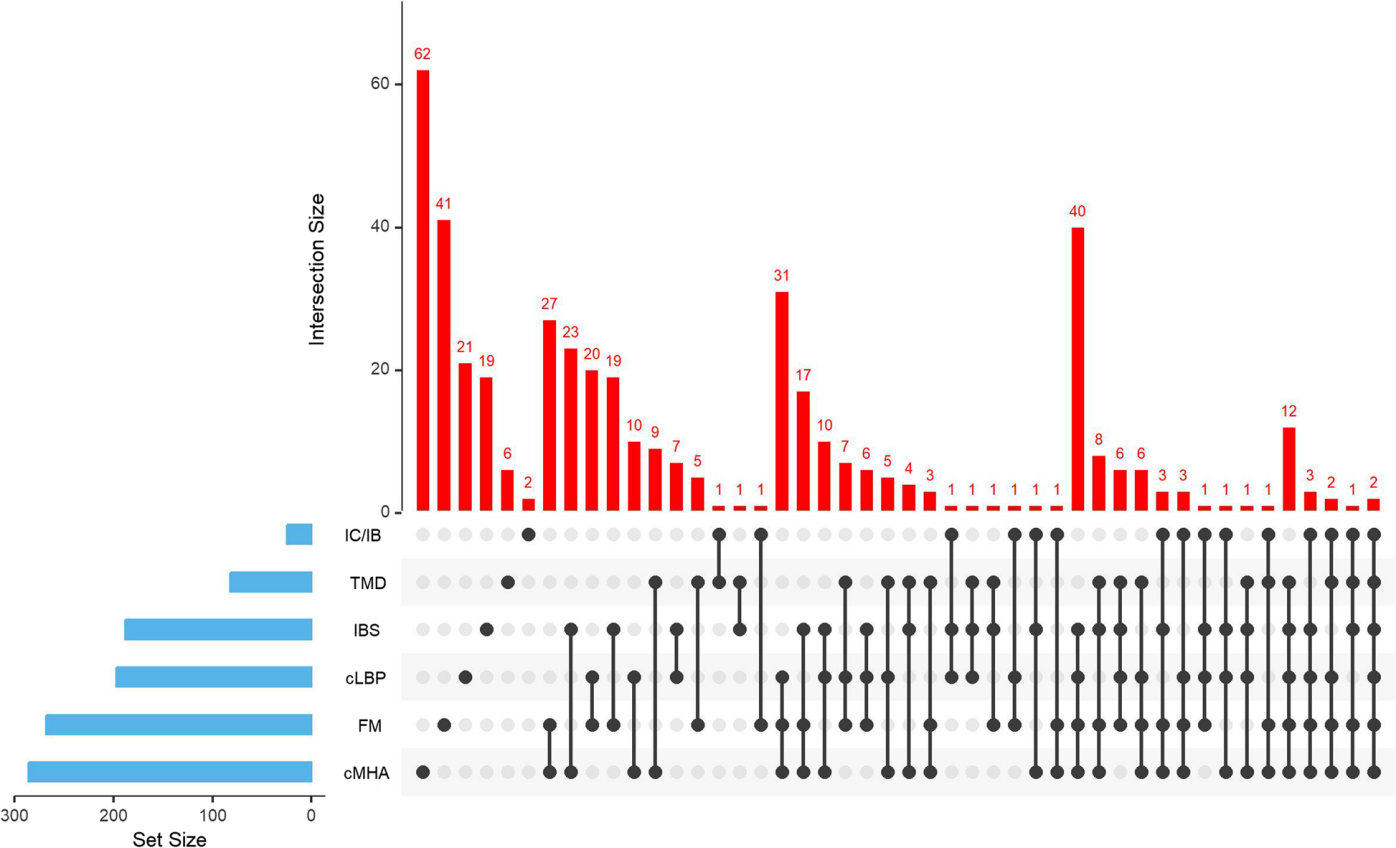
UpSet Plot for Overlapping COPCs in People with ME/CFS (*n* = 453). UpSet shows intersections in a matrix, with the rows of the matrix corresponding to the six COPC sets (from the bottom of set size: cMHA (chronic migraine/headache), FM (fibromyalgia), cLBP (chronic low back pain), IBS (irritable bowel syndrome), TMD (temporomandibular disorder), and IC/IB (interstitial cystitis/irritable bladder)), and the columns to the intersections between these sets. The size of the sets (i.e., the total number of individuals with each COPC) and of the co-occurrence among COPCs are shown as bar charts in blue and red, respectively. The set size is the same as the n for each COPCs among ME/CFS participants that was previously described in Table 2: cMHA (*n* = 286), FM (*n* = 268), cLBP (*n* = 197), IBS (*n* = 188), TMD (*n* = 82), and IC/IB (*n* = 25)

Figure 1 illustrates the co-occurrence of the six COPCs in the 453 participants with ME/CFS and at least one COPC. Of ME/CFS participants with only one COPC, cMHA was the most frequent (*n* = 62), followed by FM (*n* = 41). The combination of cMHA and FM was most common in those with two COPCs (*n* = 27). The combination of FM, cMHA, and IBS was most common in those with three COPCs (*n* = 31), and in those with four COPCs, the co-occurrence of cMHA, FM, IBS, and cLBP was the most common (*n* = 40). The most frequent co-occurrence of five COPCs, was TMD, IBS, cLBP, FM, and cMHA (*n* = 12). Finally, only two ME/CFS participants had all six COPCs.

### Impact of COPCs Comorbidities on ME/CFS

**Fig. 2:**
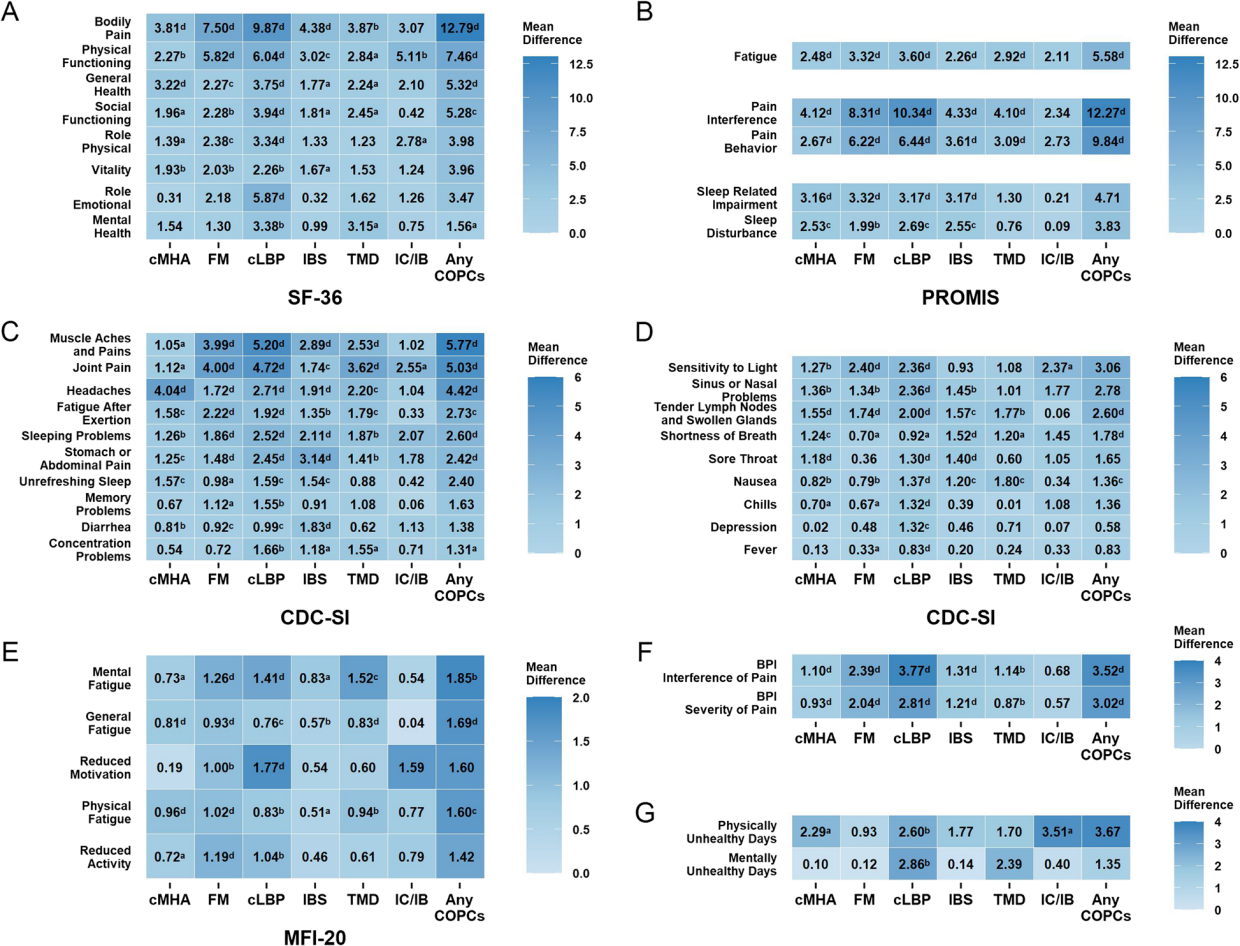
Mean Difference in Health Measures Between ME/CFS Participants With and Without Individual COPCs. Matrix graphs of health measures (A-G) by COPC comorbidities. Rows give the mean difference in specific subscale scores for ME/CFS participants with and without COPC in the column. SF-36 = 36-item Health Survey - Short Form [35]; PROMIS = Patient-Reported Outcomes Measurement Information System [29–34]; CDC-SI = CDC Symptom Inventory [26]; MFI-20 = 20-item Multidimensional Fatigue Inventory [27, 28]; BPI = Brief Pain Inventory [25]; CDC-HRQoL Unhealthy days [36]; cMHA = chronic migraine/headache; FM = fibromyalgia; cLBP = chronic low back pain; IBS = irritable bowel syndrome; TMD = temporomandibular disorder; IC/IB = interstitial cystitis/irritable bladder; COPCs = chronic overlapping pain conditions ^a^*p* < 0.05, ^b^*p* < 0.01, ^c^*p* < 0.001, ^d^*p* < 0.0001

Figure 2 depicts the mean difference in health measures between those with and without individual COPCs among the ME/CFS group. The COPC comorbidities, cLBP and FM, each had a significant impact on most health measures, most pronounced in SF-36, PROMIS, CDC-SI and BPI pain measures (Cohen’s d effect size 0.8 or larger). The largest effects were observed in BPI-IOP (cLBP: unadjusted mean difference Δ = 3.77, Cohen’s d = 1.6; FM: Δ = 2.39, d = 0.9), followed by BPI-SOP (cLBP: Δ = 2.81, d = 1.3; FM: Δ = 2.04, d = 0.9), PROMIS Pain Interference (cLBP: Δ = 10.34, d = 1.2; FM: Δ = 8.31, d = 0.9), SF-36 Bodily Pain (cLBP: Δ = 9.87, d = 1.1; FM: Δ = 7.50, d = 0.8), CDC-SI Muscle Aches and Pains (cLBP: Δ = 5.20, d = 1.1; FM: Δ = 3.99, d = 0.8), CDC-SI Joint Pain (cLBP: Δ = 4.72, d = 0.9; FM: Δ = 4.00, d = 0.8), and PROMIS Pain Behavior (cLBP: Δ = 6.44, d = 0.9; FM: Δ = 6.22, d = 0.9) (Fig. 2 and Table S3). The differences in these pain measures between those with and without individual COPCs were statistically significant in all COPCs, except for IC/IB, which was only significant for CDC-SI Joint Pain. As expected, CDC-SI Headache severity was higher in those with cMHA (Δ = 4.04, d = 0.9), and CDC-SI gastrointestinal symptoms were higher in those with IBS (Stomach or Abdominal Pain (Δ = 3.14, d = 0.8), and Diarrhea (Δ = 1.83, d = 0.6)).

While the impact of COPC comorbidities on non-pain attributes and quality of life measures was less pronounced than that on pain, statistically significant differences were still evident between ME/CFS participants with and without COPCs. Significant worsening function and symptoms with moderate effect sizes were found in SF-36 Physical Functioning (FM: Δ = 5.82, d = 0.6; cLBP: Δ = 6.04, d = 0.6; IC/IB: Δ = 5.11, d = 0.5), PROMIS Fatigue (FM: Δ = 3.32, d = 0.5; cLBP: Δ = 3.60, d = 0.5), CDC-SI Fatigue After Exertion (post-exertional malaise (PEM)) (FM: Δ = 2.22, d = 0.4; cLBP: Δ = 1.92, d = 0.4), CDC-SI Sensitivity to Light (FM: Δ = 2.40, d = 0.5; cLBP: Δ = 2.36, d = 0.5), PROMIS Sleep Related-Impairment (cMHA: Δ = 3.16, d = 0.4; FM: Δ = 3.32, d = 0.4; cLBP: Δ = 3.17,d = 0.4; IBS: Δ = 3.17, d = 0.4), Physically Unhealthy Days (cMHA: Δ = 2.29, d = 0.2; cLBP: Δ = 2.60, d = 0.3; IC/IB: Δ = 3.51, d = 0.4), and Mentally Unhealthy Days (cLBP: Δ = 2.86, d = 0.3). After adjusting for age, sex and illness duration, the COPCs impact on health measures still remain statistically significant (Table S4).

## Discussion

Our study found that COPCs pose a substantial burden for people with ME/CFS. We observed that a significant proportion (76.1%) of the participants with ME/CFS had at least one COPC comorbid condition. Of the six COPCs examined in this study, the most common comorbid conditions were chronic migraine/headache (cMHA, 48.1%), followed by fibromyalgia (FM, 45.0%), chronic low back pain (cLBP, 33.1%), and irritable bowel syndrome (IBS, 31.6%). These findings are in line with some previous research primarily focused on FM and IBS [12, 40, 41] but differ from other research [10, 13, 42, 43], especially for less studied conditions like cLBP. However, these previous studies were constrained by factors such as smaller sample sizes or geographic areas, and single clinic population. In contrast, our study benefits from a large cohort drawn from seven ME/CFS specialty clinics across the United States.

Unlike most previous research that has typically focused on one or two COPCs [44, 45], our study broadened the scope by evaluating six COPCs in both women and men with ME/CFS and two female-only COPCs among women with ME/CFS. This allowed us to assess a variety of COPCs as comorbidities or multimorbidity among people with ME/CFS. Furthermore, we also assessed the multimorbidity combinations of these conditions among ME/CFS participants. The findings provide valuable insight into the co-occurrence patterns of the six COPCs as comorbidities in people with ME/CFS that has not been explored in prior research.

Participants with ME/CFS who also had COPCs exhibited increased symptom burden and worse functioning compared to those without COPCs, particularly in measures of pain attributes. Specifically, the co-occurrence of cLBP and FM had the most substantial impact on pain among the six COPCs studied. Although research into the influence of cLBP on ME/CFS severity is sparse, existing studies have reported an escalation in pain when cLBP coexists with other COPCs. For instance, cLBP concurrent with other COPCs was found to be linked to significantly higher scores in PROMIS Pain Interference and Pain Behavior than cLBP alone [46]. Markedly higher scores of BPI Pain Severity, BPI Pain Interference, and PROMIS Pain Interference were reported in ME/CFS participants with FM compared to those with ME/CFS alone [43]. Moreover, several studies [8, 18, 19, 47] have noted that ME/CFS participants with FM scored worse on SF-36 Bodily Pain than those with ME/CFS alone. Beyond measures of pain attributes, we also observed a decline in non-pain attribute measures such as physical function, fatigue, post-exertional malaise, and sleep in ME/CFS participants with COPCs than those with ME/CFS alone. Collectively, these findings suggest that COPCs are not only prevalent among people with ME/CFS but also significantly affect their health and quality of life.

Research into mechanisms underlying interactions among COPCs and ME/CFS is needed to identify targets for therapy. Using existing pharmacologic options for COPCs [48–55] could potentially reduce symptom burden in people with ME/CFS. Consultation with pain management specialists might also be helpful [56], and noninvasive nonpharmacologic approaches have demonstrated positive outcomes in the management of COPCs [3, 49, 57]. By enhancing the efficacy of a comprehensive clinical management plan, these methods hold promise for improving patient health outcomes and quality of life in people with ME/CFS and should be weighed as part of an individualized treatment approach.

This study has some limitations that deserve attention. First, our study sample came from ME/CFS specialty clinics in the U.S. This may limit the generalizability of our findings to patients in primary care or community populations. Second, our study sample, particularly the ME/CFS group, primarily consisted of White patients so findings may not fully represent more diverse U.S. or global populations. Future research should consider community-based or diverse racial/ethnic samples for broader generalizability. Third, due to the lack of information on individual COPCs as illness controls, we were unable to compare the impact of COPCs alone (e.g., people with chronic low back pain but without ME/CFS) to ME/CFS with COPCs. Only two of the six COPCs were reported by more than 5 healthy controls. As a result, our understanding of the unique impact of individual COPCs may be limited. Future research endeavors may focus on recruiting a larger sample of ill controls, which would allow for more robust comparative analyses and contribute to a more comprehensive understanding of these conditions. Lastly, medication and treatment may influence the illness severity and functioning of people with ME/CFS and COPCs. We did not collect the medication information specifically for each COPC and were unable to factor those medications in our analysis. Future studies on COPCs treatment may help to elucidate the effect of medication on these comorbidities.

## Conclusions

In conclusion, our study highlights the importance of considering COPCs in the assessment and treatment of individuals with ME/CFS. By recognizing and addressing these comorbid conditions, clinicians may be able to improve the health and quality of life of their patients. Further research is needed to identify effective interventions for people with both ME/CFS and COPCs.

## Supplementary Information

Supplementary Material 1.

## Data Availability

Restrictions by the data custodians mean that the datasets are not publicly available or able to be provided by the authors. The program codes used in the current study are available from the corresponding author on reasonable request. Researchers wanting to access the datasets used in this study should email CDC’s ME/CFS Program (cfs@cdc.gov) and discuss next steps for the data request. The ME/CFS program data review committee will grant the access after the review and the data use agreement is finalized.

## Abbreviations

BPI: Brief Pain Inventory
BPI-IOP: Interference of Pain (IOP) in BPI
BPI-SOP: Severity of Pain (SOP) in BPI
CDC: Centers for Disease Control and Prevention
CDC-SI: CDC Symptom Inventory
cLBP: Chronic low back pain
cMHA: Chronic migraine/headache
FM: Fibromyalgia
COPCs: Chronic overlapping pain conditions
HC: Healthy controls
IBS: Irritable bowel syndrome
IC/IB: Interstitial cystitis/irritable bladder
HRQoL-14: CDC 14-item Health-Related Quality of Life
MCAM: Multi-site clinical assessment of myalgic encephalomyelitis/chronic fatigue syndrome (ME/CFS)
ME/CFS: Myalgic encephalomyelitis/chronic fatigue syndrome
MFI-20: 20-item multidimensional fatigue inventory
PR: Prevalence ratios
PROMIS: Patient-Reported Outcomes Measurement Information System
SF-36v2: Short Form Health Survey − 36-item Version 2
TMD: Temporomandibular disorder
